# Metrics for Evaluating Telemedicine in Randomized Controlled Trials: Scoping Review

**DOI:** 10.2196/67929

**Published:** 2025-01-31

**Authors:** Yuka Sugawara, Yosuke Hirakawa, Masao Iwagami, Ryota Inokuchi, Rie Wakimizu, Masaomi Nangaku

**Affiliations:** 1 Division of Nephrology and Endocrinology The University of Tokyo Tokyo Japan; 2 Department of Health Services Research, Institute of Medicine University of Tsukuba Ibaraki Japan; 3 Department of Clinical Engineering The University of Tokyo Hospital Tokyo Japan; 4 Department of Child Health and Development Nursing, Institute of Medicine University of Tsukuba Ibaraki Japan

**Keywords:** patient experience, patient-reported outcome, quality of life, quality-adjusted life year, telehealth, eHealth, mobile phone, metrics, telemedicine, systematic review, scoping review, review, telecommunications, database, health care, patient-centeredness, patient satisfaction, patient outcome, clinical parameter, cost-effectiveness, evaluation metrics, mHealth, mobile health

## Abstract

**Background:**

Telemedicine involves medical, diagnostic, and treatment-related services using telecommunication technology. Not only does telemedicine contribute to improved patient quality of life and satisfaction by reducing travel time and allowing patients to be seen in their usual environment, but it also has the potential to improve disease management by making it easier for patients to see a doctor. Recently, owing to IT developments, research on telemedicine has been increasing; however, its usefulness and limitations in randomized controlled trials remain unclear because of the multifaceted effects of telemedicine. Furthermore, the specific metrics that can be used as cross-disciplinary indicators when comparing telemedicine and face-to-face care also remain undefined.

**Objective:**

This review aimed to provide an overview of the general and cross-disciplinarity metrics used to compare telemedicine with in-person care in randomized controlled trials. In addition, we identified previously unevaluated indicators and suggested those that should be prioritized in future clinical trials.

**Methods:**

MEDLINE and Embase databases were searched for publications that met the inclusion criteria according to PRISMA-ScR (Preferred Reporting Items for Systematic Reviews and Meta-Analysis Extension for Scoping Reviews). Original, English-language articles on randomized controlled trials comparing some forms of telemedicine with face-to-face care from January 2019 to March 2024 were included, and the basic information and general metrics used in these studies were summarized.

**Results:**

Of the 2275 articles initially identified, 79 were included in the final analysis. The commonly used metrics that can be used across medical specialties were divided into the following 3 categories: (1) patient-centeredness (67/79, 85%), including patient satisfaction, workload, and quality of life; (2) patient outcomes (57/79, 72%), including general clinical parameters such as death, admission, and adverse events; and (3) cost-effectiveness (40/79, 51%), including cost assessment and quality-adjusted life year. Notably, only 25 (32%) of 79 studies evaluated all the 3 categories. Other metrics, such as staff convenience, system usability, and environmental impact, were extracted as indicators in different directions from the three categories above, although few previous reports have evaluated them (staff convenience: 8/79, 10%; system usability: 3/79, 4%; and environmental impact: 2/79, 3%).

**Conclusions:**

A significant variation was observed in the metrics used across previous studies. Notably, general indicators should be used to enhance the understandability of the results for people in other areas, even if disease-specific indicators are used. In addition, indicators should be established to include all three commonly used categories of measures to ensure a comprehensive evaluation: patient-centeredness, patient outcomes, and cost-effectiveness. Staff convenience, system usability, and environmental impact are important indicators that should be used in future trials. Moreover, standardization of the evaluation metrics is desired for future clinical trials and studies.

**Trial Registration:**

Open Science Forum Registries YH5S7; https://doi.org/10.17605/OSF.IO/YH5S7

## Introduction

### Background

Telemedicine involves any type of medical, diagnostic, or treatment-related service using telecommunication technology [1]. In Japan, the term “online medical care,” defined more specifically by the government, is used instead of telemedicine, which refers to the act of examining and diagnosing a patient, communicating the results of the diagnosis, and prescribing medical treatment in real time involving a doctor and a patient through telecommunication technology [2]. Recent developments in telecommunications technology have been remarkable. The telemedicine environment is changing with the spread of smartphones, improvements in data transmission speeds, and the development of wearable devices. Furthermore, although the use of telemedicine had been attempted earlier, it was adopted widely during the COVID-19 pandemic because of social distancing [3]. These findings indicate that the telemedicine content and environment have changed over the past few years.

The reports on the implementation of telemedicine in various medical fields are limited. Compared with standard face-to-face medical care, telemedicine reduces the risk of infection by minimizing physical contact and offers advantages to patients, such as shorter visit times, including waiting and travel times, which can improve patient satisfaction rates [4]. Ease of access to medical care provided by telemedicine may also improve disease management and clinical outcomes [5]. Telemedicine can be effective in many aspects of health care [6,7]. Therefore, the metrics that should be used to evaluate telemedicine should be carefully considered.

For evaluating disease management, using disease-specific indicators, such as hemoglobin A_1c_ (HbA_1c_) for diabetes, is necessary to determine the usefulness of telemedicine. Additionally, general indicators, including non–disease-specific indicators, are required to compare results among studies on different diseases. Telemedicine can also influence patient satisfaction and quality of life (QoL). Furthermore, the use of patient-reported outcomes (PROs) was recently proposed for the evaluation of “patient-centeredness,” the concept in which medical care is provided “for the benefit of the patient” [8].

Telemedicine affects various aspects of medical care; thus, it requires multiple measures rather than a single measure for efficacy evaluations. It is possible that indicators that have not been widely used in the past should be used in the future. A variety of metrics have been used across previous studies, and reports summarizing the general metrics used for evaluating telemedicine remain lacking.

Telemedicine does not oppose face-to-face care but rather complements it, similar to the relationship between outpatient, inpatient, and home care. However, when examining the usefulness of telemedicine, it is commonly compared with face-to-face care, which is the well-established, long-standing method of medical care. Further research into telemedicine, particularly randomized controlled trials (RCTs), is essential to generate solid evidence. To identify the metrics that should be used in future RCTs, it is essential to first understand the actual use of general metrics in past RCTs.

### Objectives

In this study, we conducted a scoping review of articles comparing telemedicine with in-person care in RCTs and summarized the metrics used to understand their usefulness. This study also aimed to identify indicators that were not thoroughly evaluated in previous clinical trials but will be useful in the future and suggest which indicators should be prioritized in future clinical trials.

## Methods

### Literature Search

This systematic literature review was conducted and reported in accordance with the PRISMA-ScR (Preferred Reporting Items for Systematic Reviews and Meta-Analysis Extension for Scoping Review) guidelines [9]. The completed PRISMA checklist is presented in Multimedia Appendix 1. MEDLINE and Embase databases were systematically searched on March 15, 2024, for peer-reviewed full papers published between January 1, 2019, and March 14, 2024. We searched only the MEDLINE and Embase databases because we assumed that most of the articles of interest (metrics used in RCTs) would be included in them. Owing to the significant advancements in telemedicine in recent years, older publications were deemed outdated, and the search was limited to the most recent publications from the past 5 years since 2019. The search string used for the search in MEDLINE contained the following terms related to telemedicine: (telemedicine OR “online medical care” OR teleconsultation OR “online consultation” OR “telemedical consultation”) AND (“randomized controlled trial”) AND (outcome OR effectiveness) AND (control OR conventional OR face-to-face). Multimedia Appendix 2 lists the search terms used in the MEDLINE and Embase databases.

The review was registered with Open Science Forum Registries (YH5S7).

### Inclusion and Exclusion Criteria

The inclusion criteria were as follows: (1) studies published between January 1, 2019, and March 14, 2024; (2) RCTs comparing some form of telemedicine with a standard of care (face-to-face care); (3) studies involving telemedicine, including online monitoring (ie, information measured using the device is automatically sent to the medical facility) or synchronous communication through telephone or video; and (4) studies involving both disease-specific metrics (eg, HbA_1c_ for diabetes) and general (ie, non–disease-specific) cross-disciplinary metrics.

The following studies were excluded: (1) protocols and proposals; (2) systematic reviews, commentaries, and scoping reviews; and (3) gray literature.

### Study Selection

Two investigators (YS and YH) independently screened the titles and abstracts of the identified studies using the Covidence systematic review software (Veritas Health Innovation). Subsequently, the investigators (YS and YH) independently reviewed the full text of the selected studies based on the inclusion and exclusion criteria. Disagreements regarding the classification of a reference were resolved by a third reviewer (MI) by conducting an additional review and confirming the final classification.

### Data Collection and Summary

Data regarding study details were extracted using the Covidence software (Veritas Health Innovation) and Microsoft Excel. One reviewer (YS) extracted the additional data from eligible studies, including the content of telemedicine and the disease-specific and general (ie, non–disease-specific) indicators for evaluating telemedicine. All extracted data were cross-checked for accuracy by a second reviewer (YH), and any discrepancies were resolved through discussion. The extracted data elements included the study title, year of publication, country, target disease or population, total sample size, type of telemedicine used, and disease-specific and general metrics used for evaluating telemedicine. A meta-analysis was deemed inappropriate because of heterogeneity in the study design, populations, and outcome measures for quantitative studies, and a narrative synthesis of quantitative study results was conducted. The evaluation metrics were categorized through the following process. Initially, all unique metrics identified by a single author (YS) were reviewed and confirmed by two additional authors (YH and MI). Following this, the 3 authors engaged in collaborative discussions to group the metrics into primary and subcategories based on shared directional perspectives. 

This study examined the actual use of metrics for evaluating telemedicine and did not focus on the results or conclusions (superiority or noninferiority of telemedicine compared with face-to-face care) of the included studies.

## Results

### Literature Search

**Figure 1:**
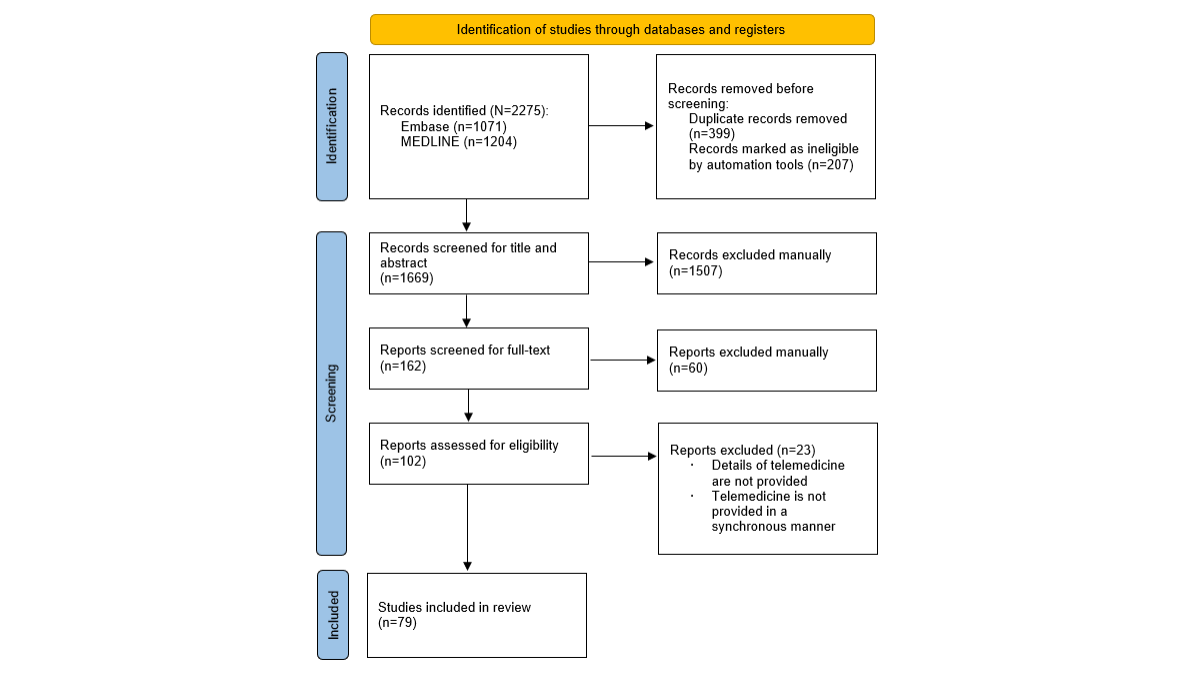
PRISMA (Preferred Reporting Items for Systematic Reviews and Meta-Analysis) 2020 flow diagram for scoping review.

Initially, 2275 studies were considered relevant according to the inclusion criteria. However, 606 duplicate studies or those marked as ineligible by automation tools were removed before screening. Furthermore, 1507 studies were excluded after screening the titles and abstracts, and 83 studies were excluded after reading the articles in greater depth during the assessment of extracted data. Finally, 79 studies were included in the analysis. The PRISMA flow diagram summarizing the article selection process is presented in Figure 1. A list of all 79 studies and their information is provided in Multimedia Appendix 3 [7,10-87].

### Study Description

Table 1 presents the characteristics of the 79 included studies. After 2020, a total of 10‐20 papers comparing telemedicine and face-to-face care in RCTs were consistently published annually. These studies were conducted and published commonly in the United States (11/79, 14%), Australia (10/79, 13%), and European countries (France: 6/79, 8%; Norway: 6/79, 8%; and: Spain 6/79, 8%), with China (5/79, 6%) and Japan (5/79, 6%) being the most common in Asia.

The most common departments in which the studies were conducted were internal medicine (39/79, 49%), psychiatry (11/79, 14%), and rehabilitation (9/79, 11%). Most dealt with outpatient care (76/79, 96%); however, a few reports dealt with telemedicine from home as an alternative to inpatient care (3/79, 4%). The specific contents were as follows: early discharge from the hospital with telemedicine versus discharge on a regular schedule (study number 57 [65]), home care under telemonitoring versus long-term hospitalization (study number 71 [79]), and initiation of treatment at home versus in the hospital (study number 74 [82]).

Regarding the targeted diseases and populations, the most common was postoperative follow-up (11/79, 14%), followed by chronic diseases, such as coronary artery disease (7/79, 9%), diabetes (6/79, 8%), and chronic obstructive pulmonary disease (4/79, 5%). The median total sample size was 106 (IQR 59‐240), with 10 and 2185 being the minimum and maximum numbers, respectively.

When examining the details of telemedicine, online medical care was the most common (69/79, 87%; Table 1), including telepsychiatry involving cognitive behavior therapy. Furthermore, online monitoring and telerehabilitation were also common practices. In several studies, telemedicine included apps for patient education and the ability to make emergency calls. Online monitoring (information measured by the device automatically sent to the medical facility) or synchronous communication through telephone or video was considered telemedicine in this review.

**Table 1:** Characteristics of the included studies (n=79).

Characteristics		Value, n (%)
**Publication year**		
	2019	9 (11)
	2020	15 (19)
	2021	18 (23)
	2022	18 (23)
	2023	12 (15)
	2024 (From January to March)	7 (9)
**Publication country^a^**		
	United States	11 (14)
	Australia	10 (13)
	France	6 (8)
	Norway	6 (8)
	Spain	6 (8)
	China	5 (6)
	Germany	5 (6)
	Japan	5 (6)
	The Netherlands	5 (6)
	United Kingdom	3 (4)
**Department^a^**		
	Internal medicine	39 (49)
	Psychiatry	11 (14)
	Rehabilitation	9 (11)
	Surgery	5 (6)
	Orthopedics	4 (5)
	Pediatrics	3 (4)
	Anesthesiology	2 (3)
	Gynecology	2 (3)
	Urology	2 (3)
**Setting**		
	Outpatient care	76 (96)
	Inpatient care	3 (4)
**Target disease or population^a^**		
	Postoperative	11 (14)
	Coronary disease	7 (9)
	Diabetes mellitus	6 (8)
	Sleep apnea	5 (6)
	Chronic obstructive pulmonary disease	4 (5)
	Mental disorders	4 (5)
	Rheumatoid arthritis	3 (4)
	Brain tumor	2 (3)
	Chronic conditions	2 (3)
	Chronic pain	2 (3)
	Heart failure	2 (3)
	Inflammatory bowel disease	2 (3)
	Insomnia	2 (3)
	Orthopedic consultation	2 (3)
	Patients with pacemakers	2 (3)
	Parkinson disease	2 (3)
	Primary care for children	2 (3)
**Detailed contents of telemedicine**		
	Online medical care^b^	69 (87)
	Telepsychiatry including cognitive-behavior therapy	10 (13)
	Online monitoring	17 (22)
	Telerehabilitation	11 (14)

^a^Two or more reports.

^b^According to the following definition by the Japanese government: the act of examining and diagnosing a patient, communicating the results of the diagnosis, and prescribing medical treatment in real time involving a doctor and a patient through telecommunication technology

### General Metrics for Evaluating Telemedicine

We identified 25 unique metrics that can be generally used (patient satisfaction, patient workload, patient time spent in medical visits, absence from work, travel distance, waiting time, QoL, patient choice of subsequent type of care, death, admission, hospital days, emergency department visits, frequency of visits or contacts, duration of visits or contacts, treatment adherence, change in diagnosis or treatment, adverse effects or safety, cost, quality-adjusted life years [QALY], staff satisfaction, staff work, staff time spent per patient, system usability, environmental impact, and feasibility for future clinical trials). A median of 4 (IQR 3-5, with a minimum of 1 and a maximum of 10) metrics were used per study. We classified commonly used metrics into three categories: (1) patient-centeredness (67/79, 85%), (2) patient outcomes (57/79, 72%), and (3) cost-effectiveness (40/79, 51%) (Table 2). Notably, 32% (25/79) evaluated all these categories.

We further subdivided the metrics into nine subcategories and examined the numbers and combinations of uses per article. Patient centeredness was divided into three categories: (1) patient satisfaction, (2) patient work, and (3) QoL. Patient outcomes were classified as (4) general clinical parameters. Cost-effectiveness was divided into two categories: (5) costs, and (6) QALY. Other metrics were categorized into three categories: (7) staff satisfaction, (8) staff work, and (9) feasibility for future clinical trial. Most studies (69/79, 87%) used two or more general metrics (ie, non–disease-specific metrics), and the median number of the nine subcategories used in each study was 3 (IQR 2-4, with a minimum of 1 and a maximum of 6) metrics. The most frequently used combinations in the nine subcategories were as follows: general clinical parameters and patient satisfaction (7/79, 9%); general clinical parameters (5/79, 6%); QoL and patient satisfaction (5/79, 6%); QoL and general clinical parameters (5/79, 6%); costs, QoL, and general clinical parameters (5/79, 6%); and costs, QALY, and QoL (5/79, 6%)

**Table 2:** General metrics for evaluating telemedicine (n=79).

Metric	Value, n (%)
Patient-centeredness	67 (85)
Patient outcomes	57 (72)
Cost-effectiveness	40 (51)
Other	40 (51)

The evaluation metrics by department are summarized in Table 3. With regard to internal medicine, psychiatry, and rehabilitation, for which a relatively large number of articles were included in this review, the utilization rate of patient outcomes was high in internal medicine (33/39, 85%) but was rather low in psychiatry (5/11, 45%) and rehabilitation (5/9, 56%). In contrast, the utilization rate of patient-centeredness was very high in psychiatry (10/11, 91%) and rehabilitation (8/9, 89%) compared with that in internal medicine (31/39, 79%).

**Table 3:** Utilization rate of general metrics by department.

Department.	Patient-centeredness, n (%)	Patient outcomes, n (%)	Cost-effectiveness, n (%)
Internal medicine (n=39)	31 (79)	33 (85)	21 (54)
Psychiatry (n=11)	10 (91)	5 (45)	7 (64)
Rehabilitation (n=9)	8 (89)	5 (56)	3 (33)
Surgery (n=5)	5 (100)	5 (100)	2 (40)
Orthopedics (n=4)	4 (100)	2 (50)	3 (75)
Pediatrics (n=3)	2 (67)	1 (33)	2 (67)
Anesthesiology (n=2)	2 (100)	1 (50)	1 (50)
Gynecology (n=2)	1 (50)	1 (50)	0 (0)
Urology (n=2)	2 (100)	2 (100)	1 (50)

### Patient-Centeredness

Patient satisfaction, work associated with medical visits, and QoL were considered metrics relevant to patient-centeredness, and these metrics were evaluated in most studies (67/79, 85%). The metrics used for evaluating patient-centeredness are summarized in Table 4.

**Table 4:** General metrics for evaluating patient-centeredness (n=67).

Metric	Value, n (%)
Patient satisfaction	41 (61)
Patient workload	17 (25)
Time spent in visit or contact	9 (13)
Absence from work	7 (10)
Travel distance	7 (10)
Waiting time	3 (4)
Quality of life	45 (67)

More than half (41/67, 61%) of the studies evaluated patient satisfaction. Table 5 presents the questionnaires used to evaluate patient satisfaction. Approximately half of the studies that addressed patient satisfaction used their own set of questions, whereas the other half used previously developed questionnaires, such as the Client Satisfaction Questionnaire [88], Telemedicine Satisfaction Questionnaire [89], and Outpatient Experiences Questionnaire [90]. In addition, 2 other studies evaluated patient experience (study numbers 48 [56] and 68 [76] in Multimedia Appendix 3), which refers to the patient’s specific “experience” of health care services. This concept is an evolution of patient satisfaction and is internationally recognized as an important quality indicator in health care [91]. Only one study evaluated “PROs.”

**Table 5:** Questionnaires used to assess patient satisfaction and quality of life (QoL). Regarding questionnaires for QoL, two reports used both the EQ-5D and SF-36^a^ QoL measures, and other two used both the EQ-5D and SF-12^b^. Others included general and disease-specific QoL measures other than EQ-5D, SF-36, and SF-12.

Questionnaires			Value, n (%)
**Patient satisfaction (n=41)**			
	Questions developed for the study	19 (46)	
	Client Satisfaction Questionnaire [88]	3 (7)	
	Telemedicine Satisfaction Questionnaire [89]	3 (7)	
	Outpatient Experiences Questionnaire [90]	2 (5)	
	Satisfad10 Questionnaire [92]	2 (5)	
	Other questionnaires from previous studies	12 (29)	
**Quality-of-life (n=45)**			
	EQ-5D-3L/5L [93,94]	26 (58)	
	SF-36^a^ [95]	9 (20)	
	SF-12^b^ [96]	4 (9)	
	Others	10 (22)	

^a^SF-36: Medical Outcomes Study (MOS) Short Form 36-Item Health Survey.

^b^SF-12: MOS Short Form 12-Item Health Survey.

Some studies (17/67, 25%) focused on work or burden associated with medical visits, including factors such as the time spent or travel distance for medical visits or contacts. Other related indicators, such as absence from work and waiting time, were also investigated in a few studies.

More than half of the studies evaluated QoL (45/67, 67%). Table 5 summarizes the metrics used to measure QoL. The most frequently used measure was the EQ-5D-3L/5L [93,94], which was used by more than half of the studies that evaluated QoL. The Medical Outcomes Study Short Form 36-Item Health Survey (SF-36) [95] and Medical Outcomes Study Short Form 12-Item Health Survey (SF-12) [96] were also frequently used. In addition to these general QoL measures, some studies also used disease-specific QoL measures.

### Patient Outcomes

When comparing telemedicine and face-to-face care in RCTs, various clinical metrics for patient outcomes, including disease-specific ones, are used to assess their superiority or noninferiority. Table 6 summarizes general metrics that appear to be applicable for evaluating various disease populations. The most commonly used general clinical parameters were admission (26/57, 46%), adherence (23/57, 40%), adverse events or safety (21/57, 37%), and frequency of visits or contacts (17/57, 30%).

**Table 6:** General metrics for evaluating patient outcomes (n=57).

Metrics	Value, n (%)
Death	10 (18)
Admission	26 (46)
Hospital days	8 (14)
Emergency department visits	10 (18)
Frequency of visits or contacts	17 (30)
Duration of visit or contact	8 (14)
Treatment adherence	23 (40)
Change in diagnosis or treatment	4 (7)
Adverse events or safety	21 (37)

### Cost-Effectiveness

The following ranges of costs were considered when evaluating cost-effectiveness: medical costs (fees for consultation, examination, medication, injection, surgery, and hospitalization), nonmedical costs (costs indirectly incurred by medical interventions, such as transportation, welfare equipment, or home improvements), and indirect costs (loss due to injury, illness, or death). The method of calculating medical costs and the range of costs included in the calculations differed across studies, partly because of differences in the reimbursement systems of the countries in which the studies were conducted and differences in perspectives (patient, insurer, or society). QALY was evaluated in 28% (11/40) of the studies that evaluated cost-effectiveness.

### Staff Convenience

Staff convenience including staff satisfaction, staff workload, and time spent per patient was evaluated in a few studies (8/79, 10%). Staff satisfaction was not frequently evaluated (4/79, 5%) compared with patient satisfaction (41/79, 52%). Similarly, fewer studies examined staff workload (5/79, 6%) than patient workload (17/79, 22%). Notably, the time spent per patient was evaluated to assess the staff work in the studies.

### System Usability

A small number of studies (study numbers 8 [18], 14 [24], and 49 [57] in Multimedia Appendix 3) have evaluated the user-friendliness of telemedicine systems using items such as usability questionnaires or the number of technical challenges encountered.

### Others

Other metrics that were not included in either of the aforementioned categories were greenhouse gas impacts (study numbers 6 [16] and 18 [27] in Multimedia Appendix 3), mental health of caregivers (study number 12 [22]), health literacy (study number 79 [87]), and self-efficacy (study number 17 [26]). Details of each are provided in Multimedia Appendix 3.

## Discussion

### Principal Findings

In this study, we conducted a scoping review of RCTs comparing telemedicine with face-to-face care published in the last 5 years and summarized the cross-disciplinary measures used in these studies. Notably, several studies used multiple measures, and metrics related to patient-centeredness, patient outcomes, and cost-effectiveness were commonly used. However, the measures used and their combinations varied across studies, and only a few studies evaluated staff satisfaction, system usability, and environmental impacts. These results highlight the requirement to standardize evaluation indicators for comparing telemedicine with face-to-face care. Although establishing evaluation measures for telemedicine is challenging owing to its multifaceted impact, the evaluation indicators can be classified into three main categories: (1) patient-centeredness, (2) patient outcomes, and (3) cost-effectiveness.

We believe that in addition to using disease-specific indicators, these three categories of general metrics should be used in telemedicine evaluations. From the papers analyzed in this study (Multimedia Appendix 3), some pertinent examples from the identified studies are as follows: Gayot et al [10] evaluated QoL for patient-centeredness, unplanned hospitalization and death for patient outcomes, and direct costs and incremental cost-effectiveness ratios for cost-effectiveness in addition to disease-specific metrics, and Mínguez Clemente et al [11] evaluated patient satisfaction for patient-centeredness; admission, hospital days, frequency of visits, and adherence for patient outcomes; and cost per patient for cost-effectiveness while using other parameters (study numbers 16 [10] and 73 [11] in Multimedia Appendix 3, respectively). Our results suggest that within a single study, at least three indicators, each belonging to a different category, should be simultaneously evaluated to ensure a comprehensive assessment of the impact of telemedicine. However, given that different diseases have different management approaches, our results indicate that the choice of metrics differs by clinical specialty (Table 3). Therefore, they need to be considered more deeply and individually in special circumstances. In particular, studies of replacing inpatient care with telemedicine were not well represented in this review and may require separate consideration.

In this study, we summarized the evaluation metrics commonly used in previous reports, identified 3 categories that should be covered, and described other 3 indicators that should be evaluated in the future. However, it did not provide a more rigorous standardization of which indicators should be used for each of the three categories. We believe that this should be discussed among the expert panel and finally decided using approaches such as the Delphi method [97].

### Patient-Centeredness

Patient-centeredness is a concept closely related to PROs [8]. Although measures of patient prognosis are relatively objective, PROs are subjective to the patient, with no intervention from a physician or measurement using instruments. However, considering such subjective evaluations as evidence is debatable [98]. Patient-centeredness is a crucial concept based on the premise that medical care is performed “for the patient,” while also emphasizing the need for standardization and clear validation. In this review, we adopted a broad perspective of patient-centeredness, considering not only studies using the term “PROs” but also the following terms as relevant: patient satisfaction, patient work associated with medical visits, and QoL.

Patient satisfaction, experience, and PROs were the main indicators used to assess patient-centeredness. Patient satisfaction is an abstract concept of “satisfaction” that also depends on individual expectations, making the standardization of measurement scales challenging and complicating the identification of specific issues from the results [99,100]. Although several questionnaires have been developed to assess patient satisfaction [88-90], their use varies across studies, and in approximately half of the studies, questions used to evaluate satisfaction were originally designed in those studies. In contrast, patient experience deals with specific “experiences” and is easier to standardize than patient satisfaction, with more convenience in identifying specific issues from the results [99,101]. PROs are even broader measures that reflect various aspects of patient health, as they are reported directly by the patient without additional interpretation by a health care professional or anyone else [8]. They may be divided into disease-specific and comprehensive PROs. Patient satisfaction, patient experience, and PROs are interrelated and cannot be studied separately. Assessing patient experience or PROs may be preferable over patient satisfaction to help identify issues.

Among the various QoL scales, EQ-5D-3L/L [93,94], SF-36 [95], and SF-12 [96] are used most commonly. Notably, even while using the disease-specific QoL assessment scales, the aforementioned general scales may be used in combination.

### Patient Outcomes

The indicators of patient outcomes can be divided into disease-specific and cross-disciplinary outcomes. In this study, disease-specific indicators are listed only in Multimedia Appendix 3 and are not tabulated as they vary across fields. Moreover, even if disease-specific indicators (eg, HbA_1c_ for diabetes) are evaluated, they may be difficult to understand for readers in other fields. Therefore, cross-disciplinary general indicators should be used in addition to disease-specific indicators. The specific parameters that should be used as general indicators depend on the severity and progression pattern of the disease but can include death, hospitalization, emergency visits, and frequency of general outpatient visits.

### Cost-Effectiveness

The most common cost-effectiveness evaluation was the calculation and comparison of the costs of telemedicine and usual face-to-face care. From the health care payer’s perspective, only medical costs are important, whereas from the patient’s perspective, only the copayment of medical costs is important. Furthermore, from a societal perspective, all medical, nonmedical, and indirect costs should be included in the analysis [102-104]. When comparing telemedicine to traditional face-to-face care, a broader spectrum of parameters must be evaluated. These encompass the costs associated with software development and hardware implementation, as well as the potential economic advantages such as reduced transportation expenses and fewer missed workdays, which are not typically considered in comparisons of in-person treatment modalities. Therefore, evaluating not only medical costs but also nonmedical and indirect costs is crucial. However, standardizing the calculation method is difficult because costs may differ depending on the reimbursement system, prices, and social environment in each country.

The QALY is a cost-effectiveness index based on QoL and survival time and is considered a common effectiveness index, even if the target diseases are different [105]. However, although various factors are associated with evaluating the cost-effectiveness of telemedicine, QALY evaluates only QoL and survival time. Therefore, this should be considered when using QALY.

### Other Metrics

In addition to the metrics mentioned earlier, future studies should also consider other metrics for evaluation. The first candidate is the metric for staff convenience. We all know that health care is “for the patient”; as noted earlier, patient-centeredness has been well investigated in many studies. On the other side of the coin, its convenience for health care professionals has not been considered important or thoroughly evaluated. However, it is an undeniable fact that the health care providers (medical professionals) are the counterparts of the health care recipients (patients), and it is easy to imagine that a system that health care providers find inconvenient is unlikely to be widely adopted. Particularly in telemedicine, health care professionals are accustomed to a well-established and easy-to-use form of care, which is face-to-face care. Therefore, evaluating whether telemedicine is easy to use for health care professionals is vital. Such information would aid in assessing how favorable telemedicine is likely to be received by health care providers when implemented in general clinical practice. Careful examination of the reasons why staff members felt telemedicine was inconvenient would also provide insight into how to improve it.

Another important factor is whether the system is easy to use, as the most important component of telemedicine is the digital equipment and internet connection. The degree of digital literacy varies among individuals, and if the system is difficult to use, telemedicine may even worsen access to health care for some people, as symbolized by the term “digital divide.” Although it has not been well evaluated so far, the evaluation metrics of system usability are also considered important for ensuring equitable access to health care.

In addition, a small number of studies evaluated the impact of greenhouse gases and environmental costs. Given the seriousness of global warming, considering a health care delivery system that is sustainable for the entire planet is also important. The Greenhouse Gas Protocol divides carbon emissions into 3 categories: scope 1, direct emissions secondary to energy use; scope 2, indirect emissions secondary to purchased electricity; and scope 3, indirect emissions [106]. Most emissions related to the health care system reportedly correspond to scope 3, which includes indirect emissions occurring as a consequence of health care activities such as disposables, equipment (medical and nonmedical), and pharmaceuticals, as it includes a wider range of emission sources than other scopes [107]. Therefore, it may be necessary to consider a wide range of targets to assess the environmental impacts of telemedicine. There is a scoping review on the inclusion of environmental impacts in evaluations of health care economics [108]. Greenhouse gas emissions, energy use, water use, and physical waste impacts were frequently used in the past, and 10 methods for assessing environmental impacts have been described. However, it remains unknown which metrics should be evaluated and in which units, creating an issue for discussion in the future.

### Limitations

Although our scoping review was thorough, it has some limitations. First, we searched only the MEDLINE and Embase databases for peer-reviewed, full-text articles, although we assumed that most of the articles in our scope would be included within them because we focused on the metrics used in RCTs. Second, only papers published in 2019-2024 were included because the content of telemedicine has changed rapidly with technological advancements and the emergence of COVID-19, and it is unclear whether it is appropriate to apply the content of the distant past to current analyses. This limited the scope of the search and emphasized the most recent data. Third, a narrative synthesis is a secondary analysis of data that focuses on the interpretations presented by the authors of original papers and is not based on primary data. Furthermore, our findings represent an interpretation of the data and should be viewed as a heuristic theory.

### Conclusions

In studies comparing telemedicine with face-to-face care from 2019 to 2024, the most commonly used metrics were patient-centeredness, patient outcomes, and cost-effectiveness. These are important indicators of telemedicine and should be evaluated simultaneously in future clinical studies. In addition, our study also indicates that staff satisfaction could be an important evaluation metric for future clinical trials.

## Appendix

Multimedia Appendix 1 PRISMA-ScR (Preferred Reporting Items for Systematic reviews and Meta-Analyses extension for Scoping Reviews) checklist.

Multimedia Appendix 2 Search terms used for the MEDLINE and Embase databases.

Multimedia Appendix 3 List of all the 79 papers and their information.

## Abbreviations

HbA1c: hemoglobin A1c
IQR: interquartile range
PRISMA-ScR: Preferred Reporting Items for Systematic Reviews and Meta-Analysis Extension for Scoping Reviews
PRO: patient-reported outcome
QALY: quality-adjusted life year
QoL: quality of life
RCT: randomized control trial
SF-12: Medical Outcomes Study Short Form 12-Item Health Survey
SF-36: Medical Outcomes Study Short Form 36-Item Health Survey

## Data Availability

All data analyzed during this study are included in the article and its supplementary files.
