# Supplementary material for: Metrics for Evaluating Telemedicine in Randomized Controlled Trials: Scoping Review
Source: J Med Internet Res. 2025 Jan 31;27:e67929. doi: 10.2196/67929 (PMC11829184; doi:10.2196/67929)
Supplement: Multimedia Appendix 2 [file jmir_v27i1e67929_app2.pdf]

MEDLINE: (telemedicine OR "online medical care" OR teleconsultation OR "online consultation" OR "telemedical consultation") AND ("randomized controlled trial") AND (outcome OR effectiveness) AND (control OR conventional OR face-to-face)
1st January, 2019 to 14th March, 2024

Embase: #1 (('telemedicine' or 'online medical care' or 'teleconsultation' or 'online consultation' or 'telemedical consultation') and 'randomized controlled trial' and ('outcome' or 'effectiveness') and ('control' or 'conventional' or 'face to face')).mp. [mp=title, abstract, heading word, original title, device manufacturer, device trade name, keyword heading word, floating subheading word, candidate term word]
#2 limit 1 to dc="20190101 - 20240314"
